# First person – Sarita Hebbar and Malte Lehmann

**DOI:** 10.1242/bio.058519

**Published:** 2021-01-27

## Abstract

First Person is a series of interviews with the first authors of a selection of papers published in Biology Open, helping early-career researchers promote themselves alongside their papers. Sarita Hebbar and Malte Lehmann are co-first authors on ‘Mutations in the splicing regulator Prp31 lead to retinal degeneration in *Drosophila*’, published in BiO. Sarita is a post-doctoral fellow in the lab of Elisabeth Knust at the Max Planck Institute of Molecular Cell Biology & Genetics, investigating how the same metabolic pathways regulate temporally distinct processes (in morphogenesis and later in tissue homoestasis). Malte is a post-doctoral researcher and physician in the lab of R. G. Kühl and A. G. Siegmund at Charité, Universitätsmedizin Berlin, investigating the mechanisms behind inflammation in Inflammatory Bowel Disease patients.


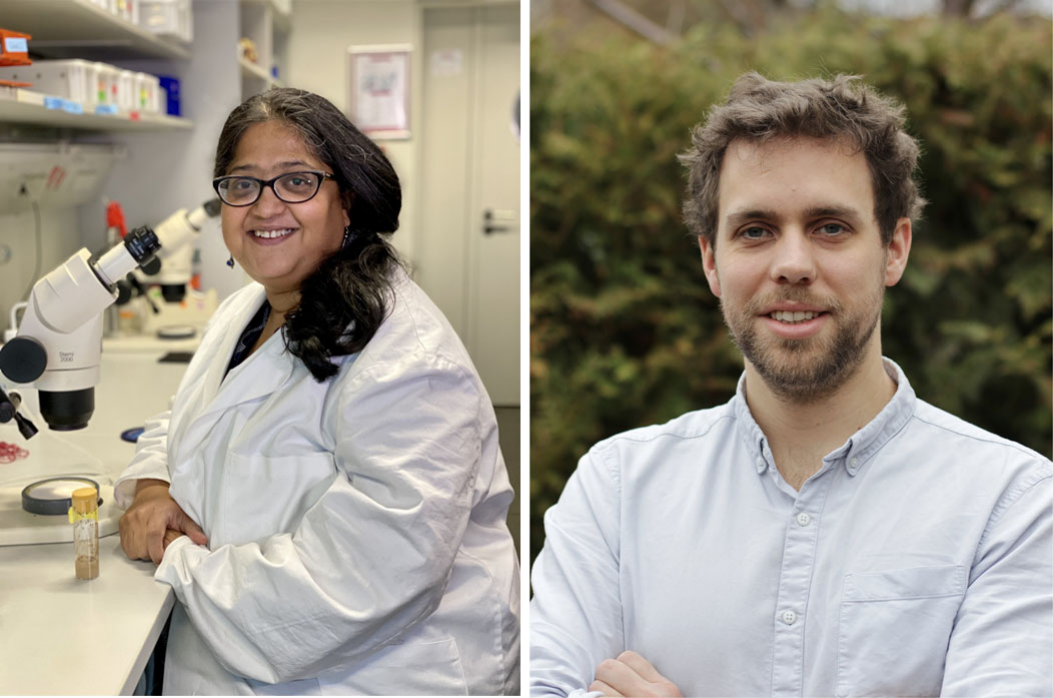


**Sarita Hebbar and Malte Lehmann**

**What is your scientific background and the general focus of your lab?**

**SH:** I am a biologist. I am interested in the cellular and molecular events that lead to the gradual loss of healthy cells as seen in ageing and in degenerative diseases. In the Knust laboratory, our focus is on identifying mechanisms that underlie retinal degeneration.

**ML:** I studied medicine and some aspects of this manuscript were part of my MD thesis. Currently, I am a physician-researcher with an interest in inflammation.

**How would you explain the main findings of your paper to non-scientific family and friends?**

We have generated an experimentally accessible model of a human disease called Retinitis Pigmentosa (RP). RP is the term for a collection of retinal diseases causing blindness in adults. To date, mutations in over 40 genes have been linked to RP. We have generated a model of a specific RP, RP11, with defined mutations in the gene *Prpf31* in the fruit fly, *Drosophila melanogaster*. Flies carrying a mutation in this gene become blind. Although the fly retina and the human retina are structurally diverse, their building blocks and many genes are conserved in evolution. Therefore, the fly can be considered as a living test tube. By modeling diseases in flies, questions of disease onset and progression with age and their cellular basis can be addressed in a living organism, which is not possible in cultured cells.

The gene *Prpf31* is expressed in many tissues in our body and plays a role in generating appropriately sized and coding gene products (called messenger RNA or mRNA). mRNA carries the instruction for the generation of proteins which carry out different functions in a cell. The process of cutting a longer RNA into smaller pieces and joining these to coding mRNAs is called splicing and Prpf31 is part of the cellular splicing machinery. A disruption of splicing will result in mRNAs that encode proteins with no or defective functions.

Here, we describe the generation and characterization of flies with defined mutations in *Prpf31*. These mutations recapitulate the retinal degeneration associated with RP11 in humans. This allowed us to identify a connection between mutation in *Prpf31* and the cellular consequences leading to retinal degeneration. One of these consequences is an increased level of the protein Opsin, which is instrumental for vision.

“[We] identify a connection between mutation in *Prpf31* and the cellular consequences leading to retinal degeneration.”

**What are the potential implications of these results for your field of research?**

Our findings have opened avenues for future research. This model can be used to investigate the general roles of Prpf31, for instance in tissues other than the eye. It will help us understand why the eye is especially vulnerable to splicing defects as seen in RP conditions. A second possibility is to further investigate the regulation of the synthesis of Opsin. Opsin-related perturbations are characteristic hallmarks of other retinal diseases as well. This raises the question if it is possible to increase/decrease Opsin by manipulating its biosynthesis and without affecting other cellular processes?

“This model can be used to investigate the general roles of Prpf31, for instance in tissues other than the eye.”

**What has surprised you the most while conducting your research?**

Although we are aware of it, we were still surprised with the level of conservation of gene function between flies and humans. Apart from the molecular similarity of the gene, the disease manifestation in mutant flies (described here) recapitulates many features of the human disease. For instance, inheriting just one copy of the mutated gene is sufficient for manifestation of the disease in flies as in humans. Likewise, the genetic background appears to be very crucial in the severity of the disease. This is also similar to the situation in humans wherein the same genetic mutation is associated with a varying degree of severity in the disease onset/progression.

**What, in your opinion, are some of the greatest achievements in your field and how has this influenced your research?**

Our understanding of the genetic basis of development of an organism is largely due to the pioneering work with *Drosophila melanogaster*. In the past few decades, it has increasingly been used as a model system for human diseases. This was made possible only because of the generation of sophisticated genetic tools, the community spirit of researchers working with flies, the open access to genetic sequences and tools, and platforms such as the Flybase or the Bloomington Stock Centre. Ultimately, all of the above were instrumental in enabling us to generate an experimentally useful disease model of RP11.

**Figure:**
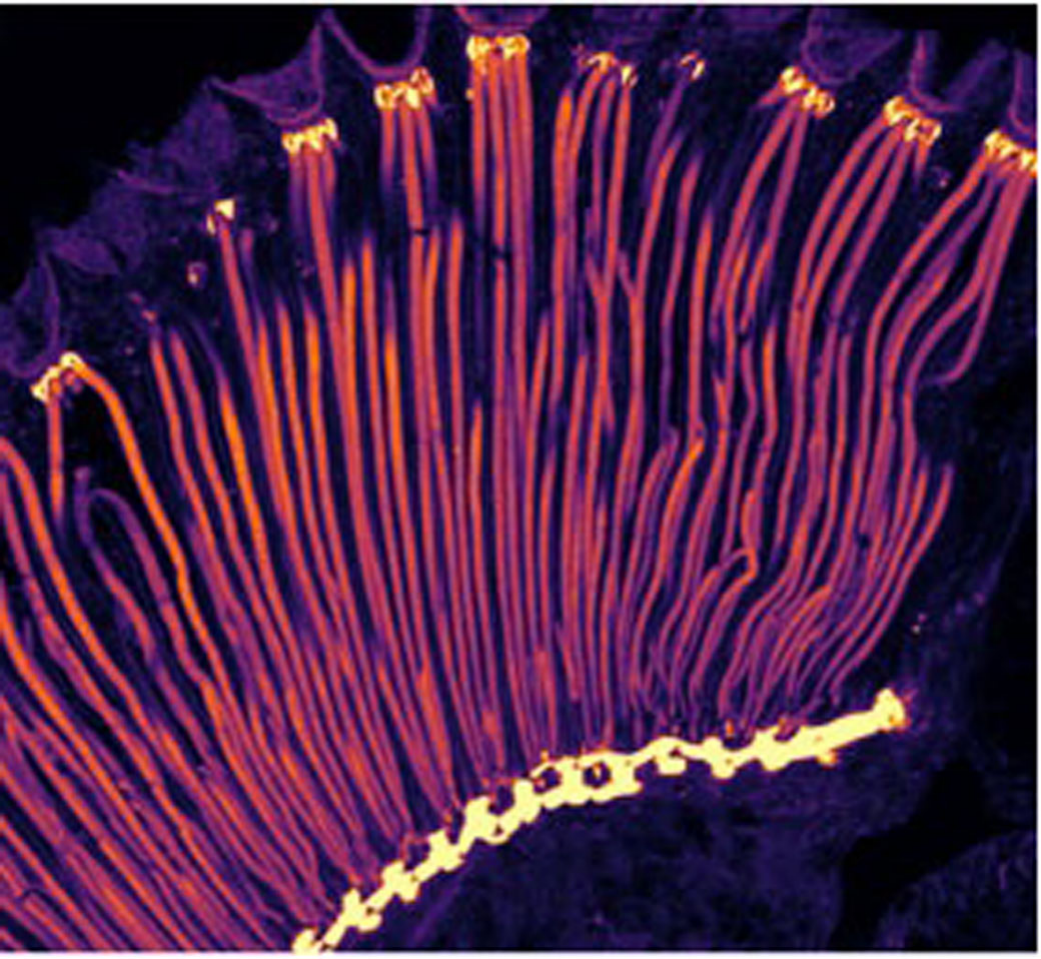
**A confocal image of a longitudinal section of a fly eye that is labelled with a fluorescent stain for Filamentous-Actin (F-actin).** F-actin is enriched (pseudocolored as yellow) in the proximal (top) and distal (bottom) regions of the retina. The compound nature of the fly eye is visible by the multiple repeated bundles of long photoreceptor cells. Also visible are F-Actin enriched (magenta) compartments of phototransduction (called the rhabdomere) in photoreceptor cells is seen.

**What changes do you think could improve the professional lives of early-career scientists?**

**SH:** I have the following suggestions: (a) age/experience in research needs to be given the value it deserves from recruitment committees/funding agencies. Many experienced post-doctoral fellows are left without any options, for job applications and funding applications, due to maximum age restrictions despite their contribution and potential. (b) Funding/support of basic research. Funding bodies should continue to have an appreciation of traditional and powerful ways to address underlying mechanisms of cellular processes. We have been very fortunate to receive funding from the Max Planck Society that supports basic research.

**ML:** I was lucky to get such support from Elisabeth Knust as a young scientist coming from the medical field. However, there is a great need to extend the possibilities for medical students and young physicians to get protected time away from the clinic to do good quality research medical students and young physicians to get protected time away from the clinic to do good quality research.

**What's next for you?**

**SH:** I will continue using the powerful system of *Drosophila melanogaster* to address important questions in biology.

**ML:** I am training as a physician in internal medicine/gastroenterology. Recently I started using imaging mass cytometry to investigate the effect of COVID-19 on the small intestine and mechanisms behind the development of fistulas in Crohn's disease.
